# Aspartate Aminotransferase to Platelet Index (APRI) as a Prognostic Factor for Overall Survival After TARE Y90 Resin Microspheres in Primary Liver Cancer Patients: Results from the European, Prospective Observational Study CIRT

**DOI:** 10.1007/s00270-026-04483-7

**Published:** 2026-05-27

**Authors:** Niklaus Schaefer, Dirk Arnold, Niels de Jong, Milan Geyer, Maria Urdaniz, Thomas Helmberger

**Affiliations:** 1 Service de médecine nucléaire et imagerie moléculaire, centre hospitalier universitaire vaudois (CHUV), Rue du Bugnon 46, 1011 Lausanne, Switzerland; 2 Oncology and Hematology, Asklepios Tumorzentrum Hamburg, AK Altona, Paul-Ehrlich-Str. 1, 22763 Hamburg, Germany; 3 P+F Products and Features GmbH, Bösendorferstraße 5/3, 1010 Vienna, Austria; 4 Next Research GmbH, Cardiovascular and Interventional Radiological Society of Europe, Neutorgasse 9, 1010 Vienna, Austria; 5 Department of Radiology, Neuroradiology and Minimal-Invasive Therapy, Klinikum Bogenhausen, Englschalkinger Str. 77, 81925 Munich, Germany

**Keywords:** Radioembolization, Transarterial radioembolization, Yttrium-90, SIR-spheres, TARE, APRI, AST to platelet index

## Abstract

**Purpose:**

To evaluate the prognostic value of the aspartate aminotransferase-to-platelet ratio index (APRI) for overall survival (OS) after transarterial radioembolization (TARE) in patients with intrahepatic cholangiocarcinoma (ICC) and hepatocellular carcinoma (HCC) from the prospective study CIRT (CIRSE study on SIR-Spheres Therapy).

**Materials and Methods:**

Adult patients treated with TARE Y-90 were included. Baseline, laboratory, treatment, and follow-up data were collected prospectively. Receiver operating characteristic analysis was used to determine optimal APRI cut-offs. OS from the date of TARE was analysed using Kaplan–Meier. Propensity score matching and multivariable Cox regression analyses were performed.

**Results:**

103 ICC and 377 HCC patients were analysed. Most patients had ECOG 0 (58.3%/61.3%) or 1 (31.1%/31.8%) in the ICC and HCC groups, respectively. Median APRI was 0.5 (IQR: 0.3–0.8) in ICC and 0.8 (IQR: 0.4–1.4) in HCC. Higher APRI values were associated with significantly shorter OS in both cohorts using different thresholds (ICC: APRI > 0.4; HCC: APRI > 0.7). These associations remained significant after propensity score matching and multivariable analysis. APRI was an independent prognostic factor for OS in both ICC (95% CI 1.25–5.23; *p* = 0.010) and HCC (95% CI 1.04–2.06; *p* = 0.028). The identified APRI cut-offs were associated with survival differences when body surface area (BSA) and modified BSA dosimetry were used, but not with partition model dosimetry.

**Conclusion:**

APRI may be a promising, easily accessible prognostic biomarker for OS after TARE in ICC and HCC patients. Its prognostic value may be influenced by tumour type and counteracted by dosimetry method, supporting further investigation on this topic.

*Trial registration* NCT02305459

**Graphical Abstract:**

**Supplementary Information:**

The online version contains supplementary material available at https://doi.org/10.1007/s00270-026-04483-7.

**Figure:**
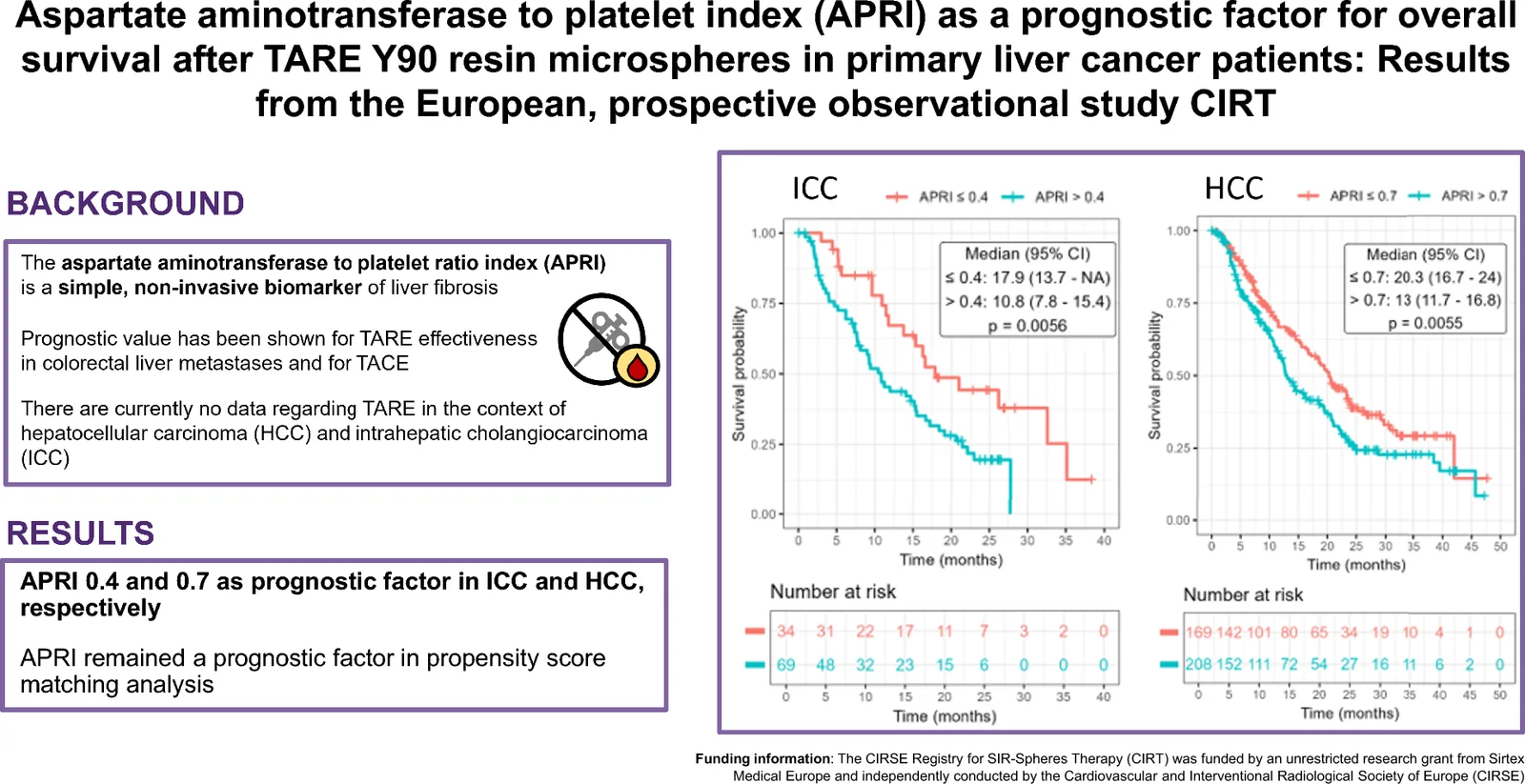

## Introduction

Transarterial radioembolization (TARE) is a well-established treatment for hepatocellular carcinoma (HCC) and intrahepatic cholangiocarcinoma (ICC) [1–3]. Given that patients often undergo multiple procedures owing to the combined use of TARE and the complexity of liver cancer management, it is essential for physicians to implement decision strategies that maximise positive patient outcomes—including patient’s safety—and patient’s quality of life [4–6]. Also, patient selection for TARE remains challenging, partly because liver function metrics do not fully capture the complexity of hepatic reserve required to guide treatment decisions. In this context, identifying prognostic factors for overall survival (OS) after TARE Y90 becomes important in assessing whether TARE is likely to achieve the desired outcomes.

The aspartate aminotransferase to platelet ratio (APRI) is a well-established biomarker for liver fibrosis and cirrhosis [7, 8]. APRI is calculated by assessing the ratio of serum aspartate aminotransferase (AST) levels to platelet count, making it an easily accessible and non-invasive test to assess liver damage. While some studies have described the connection between APRI and treatment outcomes for TACE in HCC patients [9–11], no studies have examined its potential as prognostic factor for OS after TARE. Existing metrics such as the Child–Pugh score have recognised limitations in discriminating liver function, and there is growing evidence supporting the value of additional objective markers—including fibrosis-based indices such as APRI—to improve pre-treatment assessment [12, 13]

The Cardiovascular and Interventional Radiological Society of Europe (CIRSE) conducted CIRT (CIRSE study on SIR-Spheres Therapy, NCT02305459) (SIR-Spheres®, Sirtex Medical Limited, St. Leonards, NSW, Australia), and in 2022 published its cohort of patients with colorectal cancer liver metastases (CRLM) treated with TARE [14]. The analysis identified APRI ≤ 0.4 as an independent prognostic factor for improved OS (*p* < 0.0001), progression-free survival (PFS) (*p* = 0.0416) and hepatic PFS (hPFS) (*p* = 0.0207), among other prognostic factors. Building on these findings, the aim of the present analysis is to investigate the role of APRI as prognostic factor for OS after TARE in the HCC and ICC cohorts.

## Methods

### Study Design and Setting

CIRT is a prospective, observational registry of patients with primary and secondary liver tumours treated with TARE yttrium-90 (Y90) resin microspheres in Europe. The methodology of CIRT was published elsewhere [15]. In the present analysis, primary liver cancer (HCC and ICC) cohorts of CIRT are included.

### Patient and Site Selection

Adult primary liver cancer (HCC and ICC) patients treated with TARE with Y90 resin microspheres were included in this analysis. There were no specific patient exclusion criteria. All patients in CIRT signed an informed consent form.

Sites with a minimum of TARE experience (i.e., ≥ 10 TARE treatments in the previous 12 months and a career history of at least 40 cases) were invited to participate in the study.

Treatments were performed following routine clinical practices of the participating sites. Study procedures were performed in accordance with the ethical standards of the applicable institutional and/or national ethics committees and followed the 1975 Helsinki Declaration.

### Endpoints and Objectives

The primary aim of this analysis is to evaluate the prognostic value of APRI for OS after TARE in primary liver cancer (HCC and ICC) patients.

### Assessments

Baseline, treatment and follow-up data were collected using an electronic data capture system (ConexSys Inc Lincoln, RI, United States) hosted on a local secured server in Vienna maintained by ITEA (good Information Technology, good idEAs), Vienna, Austria. Follow-up assessments were collected every three months for a minimum of 2 years. OS was calculated from the day of the TARE treatment until day of death.

Receiver operating characteristic (ROC) curve analysis was performed to determine optimal APRI cut-offs for ICC and HCC with OS as endpoint, the cut-offs 0.4 (sensitivity: 0.77, specificity: 0.43) for ICC and 0.65 (sensitivity: 0.62, specificity 0.48) and 0.77 (sensitivity: 0.61, specificity: 0.52) for HCC were found. In addition, previously published cut-offs (0.4, 0.7, 1.0 and 2.0) [16] were evaluated in time-to-event-analysis. Cut-offs of 0.4 and 0.7 (sensitivity: 0.60, specificity: 0.51) for ICC and HCC respectively were selected to ensure comparability with existing literature while still falling within the best performing cut-offs of our ROC analysis.

Further details on the statistical analyses, including variables used for propensity score matching, are provided in the supplementary material.

## Results

Baseline and disease characteristics for the ICC (n = 103) and HCC (n = 377) cohorts are summarised in Table 1. Additional information is available in Supplementary Table 1.

**Table 1:** Patient and disease characteristics by indication

Category	Subcategory	Cholangiocarcinoma	Hepatocellular carcinoma
*Total*			
		103	377
*Age*			
	n (%)	103 (100%)	377 (100%)
	Median (IQR)	63.0 (56.5 to 72.0)	68.0 (60.0 to 74.0)
*Gender*			
	n (%)	103 (100%)	377 (100%)
	Female	46 (44.7%)	68 (18%)
	Male	56 (54.4%)	305 (80.9%)
	Unknown	1 (1%)	4 (1.1%)
*ECOG*			
	n (%)	100 (97.1%)	377 (100%)
	0	60 (58.3%)	231 (61.3%)
	1	32 (31.1%)	120 (31.8%)
	2	7 (6.8%)	22 (5.8%)
	3	1 (1%)	4 (1.1%)
*BCLC*			
	n (%)	–	377 (100%)
	A–Early stage	–	51 (13.51%)
	B–Intermediate stage	–	198 (52.5%)
	C–Advanced stage	–	125 (33.2%)
	D–Terminal stage	–	3 (0.8%)
*Prior hepatic procedures*			
	n (%)	103 (100%)	377 (100%)
	No	68 (66%)	205 (54.4%)
	Yes	35 (34%)	172 (45.6%)
*Prior systemic therapy*			
	n (%)	103 (100%)	377 (100%)
	No	43 (41.7%)	340 (90.2%)
	Yes	60 (58.3%)	37 (9.8%)
*Extra hepatic disease*			
	n (%)	103 (100%)	377 (100%)
	No	69 (67%)	342 (90.7%)
	Yes	34 (33%)	35 (9.3%)
*AST* (U/L)			
	n (%)	103 (100%)	377 (100%)
	Median (IQR)	39.0 (27.8 to 58.0)	47.0 (31.0 to 72.0)
*Platelets* (*10^9/L)			
	n (%)	103 (100%)	377 (100%)
	Median (IQR)	194.0 (146.0 to 262.0)	158.0 (110.0 to 231.0)
*APRI*			
	n (%)	103 (100%)	377 (100%)
	Median (IQR)	0.5 (0.3 to 0.8)	0.8 (0.4 to 1.4)
*Albumin* (g/dl)			
	n (%)	93 (90.3%)	354 (93.9%)
	Median (IQR)	3.9 (3.4 to 4.2)	3.7 (3.4 to 4.1)
*Bilirubin* (µmol/L)			
	n (%)	103 (100%)	376 (99.7%)
	Median (IQR)	10.4 (7.4 to 14.8)	14.0 (9.0 to 19.2)
*ALBI*			
	n (%)	93 (90.3%)	353 (93.6%)
	A1	51 (49.5%)	130 (34.5%)
	A2	41 (39.8%)	209 (55.4%)
	A3	1 (1%)	14 (3.7%)

IQR, interquartile range; ECOG, Eastern Cooperative Oncology Group; BCLC, Barcelona Clinic Liver Cancer; AST, Aspartate Aminotransferase; APRI, Aspartate Aminotransferase to Platelet Index; ALBI, Albumin-Bilirubin

### ICC

**Fig. 1:**
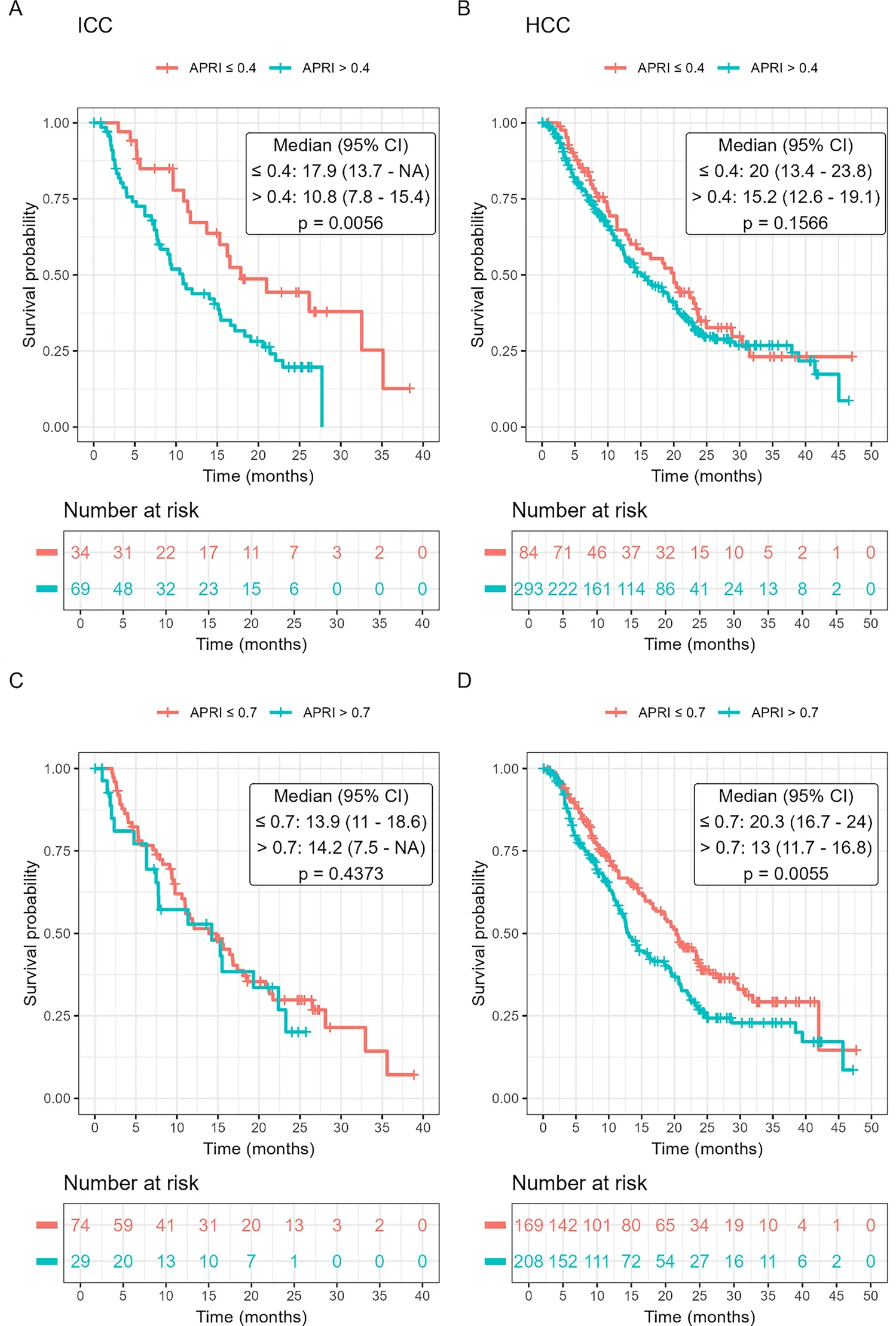
Kaplan–Meier curves on the effect of APRI cut-offs on survival. **A** ICC APRI cut-off 0.4. **B** HCC APRI cut-off 0.4. **C** ICC APRI cut-off 0.7. **D** HCC APRI cut-off 0.7. Overall Survival of ICC (A and C) and HCC (C and D) with APRI cut-offs 0.4 (A and B) and 0.7 (C and D)

Median APRI was 0.5 (interquartile range [IQR]: 0.3–0.8). More than half of the patients (58.3%) had received prior systemic therapy (Table 1). 34 (33%) and 69 (67%) patients had APRI ≤ 0.4 and APRI > 0.4, respectively; and 74 (72%) and 29 (28%) had APRI ≤ 0.7 and APRI > 0.7, respectively (Fig. 1). For individuals with ICC, APRI > 0.4 showed a significant correlation with shorter OS (*p* = 0.0056; Fig. 1A).

After propensity score matching, a multivariable analysis was performed to assess the potential impact of other factors (Table 2). In the ICC cohort, compared to single tumours, having 2–5 tumours (1.04–5.19, *p* = 0.039), > 5 tumours (1.09–5.71, *p* = 0.031) and uncountable tumours (1.57–6.86, *p* = 0.002) were correlated with shorter OS.

**Table 2:** Multivariable analysis by indication

OS variable		Value or n (%)	HR (univariable, 95% CI)	HR (multivariable, 95% CI)
Intrahepatic cholangiocarcinoma (ICC)				
*Tumour burden (nodules)*				
	1	35 (35.0)	-	-
	2–5	23 (23.0)	2.37 (1.14–4.91, *p* = 0.021)	2.32 (1.04–5.19, *p* = 0.039)
	> 5	16 (16.0)	3.53 (1.68–7.43, *p* = 0.001)	2.49 (1.09–5.71, *p* = 0.031)
	Uncountable	26 (26.0)	2.54 (1.31–4.92, *p* = 0.006)	3.28 (1.57–6.86, *p* = 0.002)
*Portal vein*				
	Patent	83 (83.0)	–	–
	Segmental thrombosis	9 (9.0)	1.00 (0.43–2.33, *p* = 0.999)	0.80 (0.32–1.98, *p* = 0.624)
	Lobar thrombosis	4 (4.0)	0.66 (0.16–2.70, *p* = 0.560)	0.45 (0.22–0.90, *p* = 0.025)
	Main thrombosis	4 (4.0)	4.21 (1.44–12.29, *p* = 0.009)	3.58 (1.71–7.49, *p* = 0.001)
*APRI*				
	≤ 0.4	33 (33.0)	–	–
	> 0.4	67 (67.0)	2.20 (1.24–3.90, *p* = 0.007)	2.55 (1.25–5.23, *p* = 0.010)
*Creatinine (mg/dl)*				
	Mean (SD)	1.0 (1.0)	1.20 (1.00–1.44, *p* = 0.052)	1.13 (1.00–1.27, *p* = 0.052)
Hepatocellular carcinoma (HCC)				
*Extra-hepatic disease at first treatment*				
	No	339 (90.6)	-	-
	Yes	35 (9.4)	1.92 (1.27–2.89, *p* = 0.002)	1.49 (0.97–2.28, *p* = 0.066)
*Liver tumour location*				
	Bilobar	129 (34.5)	–	–
	Left	49 (13.1)	0.54 (0.35–0.84, *p* = 0.006)	0.52 (0.27–1.02, *p* = 0.059)
	Right	196 (52.4)	0.49 (0.37–0.66, *p* < 0.001)	0.50 (0.32–0.79, *p* = 0.003)
*Portal Vein*				
	Patent	252 (67.4)	–	–
	Segmental thrombosis	76 (20.3)	1.06 (0.76–1.49, *p* = 0.720)	1.36 (0.93–1.98, *p* = 0.113)
	Lobar thrombosis	30 (8.0)	1.41 (0.86–2.32, *p* = 0.169)	1.49 (0.76–2.92, *p* = 0.250)
	Main thrombosis	16 (4.3)	2.46 (1.39–4.34, *p* = 0.002)	2.40 (1.41–4.09, *p *= 0.001)
*Dosimetry model*				
	BSA/mBSA	202 (54.3)	–	–
	Partition	170 (45.7)	0.52 (0.39–0.69, *p* < 0.001)	0.70 (0.44–1.10, *p* = 0.125)
*APRI*				
	≤ 0.7	166 (44.4)	–	–
	> 0.7	208 (55.6)	1.44 (1.10–1.89, *p* = 0.008)	1.46 (1.04–2.06, *p* = 0.028)

**ICC:** Samples in model = 98, Concordance = 0.703 (SE = 0.036), R^2^ = 0.263, Likelihood ratio test = 29.929 (df = 8, *p* < 0.001)

**HCC:** Samples in model = 368, Concordance = 0.672 (SE = 0.022), R^2^ = 0.167, Likelihood ratio test = 67.193 (df = 8, *p* < 0.001)

OS, overall survival; HR, hazard ratio; CI, confidence interval; SD, standard deviation; APRI, Aspartate Aminotransferase to Platelet Index; BSA, body surface area; SE, standard error; df, degrees of freedom

To evaluate the differences between body surface area (BSA) and modified BSA (mBSA) as a broad strategy for calculating approximate dose and personalised models, dosimetry methods were assessed for the different cut-offs using both the empirical BSA/mBSA method and the more personalised partition model. OS differed by APRI threshold when BSA/mBSA was used, with higher APRI (> 0.4) associated with poorer outcomes (Supplementary Fig. 1A). In contrast, under partition model OS differences were not shown (Supplementary Fig. 1B). These results should be interpreted with caution based on the strong correlation between dosimetry and tumour spread (Supplementary Table 2).

Sensitivity analyses including ALBI grade as a marker of liver function were performed. In ICC patients, APRI > 0.4 remained independently associated with worse OS (HR 2.58, 95% CI 1.42–4.71, *p* = 0.002), indicating that the prognostic value of APRI was not explained by liver function alone (Supplementary Tables 3 and 4).

### HCC

Half of the patients (52.5%) had intermediate stage BCLC-B, and 9.8% had received prior systemic therapy. Median APRI was 0.8 (IQR: 0.4–1.4) (Table 1). 84 (22.3%) and 293 (77.7%) patients had APRI ≤ 0.4 and APRI > 0.4, respectively; and 169 (44.8%) and 208 (55.2%) had APRI ≤ 0.7 and APRI > 0.7, respectively (Fig. 1). APRI > 0.7 showed a significant correlation with shorter OS compared to patients with APRI ≤ 0.7 (*p* = 0.0055; Fig. 1D).

Results from the multivariable analysis showed that extrahepatic disease (0.97–2.28, *p* = 0.066) and main PVT (1.41–4.09, *p* = 0.001) were correlated with shorter OS. Right-lobe tumour location (0.32–0.79, *p* = 0.003) was associated with longer survival in the HCC cohort (Table 2).

Similar to the ICC group, OS differed by APRI threshold when BSA/mBSA was used, with higher APRI (> 0.7) associated with poorer outcomes (Supplementary Fig. 1C). These survival differences were not observed under partition model (Supplementary Fig. 1D).

In HCC patients the association between APRI > 0.7 and OS lost statistical significance after adjustment for ALBI grade (HR 1.21, 95% CI 0.80–1.83, *p* = 0.361), suggesting that the effect of APRI in this population may largely reflect underlying liver dysfunction (Supplementary Table 4).

## Discussion

In both ICC and HCC, lower APRI values were associated with significantly longer OS. Using the proposed cut-offs (0.4 for ICC and 0.7 for HCC), significant survival differences we observed in both groups (Fig. 1). While specific APRI cut-offs connected to OS after TARE have not been reported, a meta-analysis indicated that thresholds of 0.7 and 1.0 are correlated with significant fibrosis and cirrhosis, respectively in HCC [16]. The original study defining APRI reported thresholds of 0.88 and 0.94 for significant fibrosis and cirrhosis, respectively [7].

In this analysis, the prognostic role of APRI was confirmed in multivariable Cox analysis after propensity score matching (Table 2). After balancing for relevant confounders—including age, ECOG status, prior hepatic procedures, and creatinine—APRI retained its independent association with survival. In HCC, APRI > 0.7 remained significantly associated with a 46% increase of risk of death (HR = 1.46, *p* = 0.028), and in ICC, APRI > 0.4 conferred more than twice the risk (HR = 2.55, *p* = 0.010).

In patients treated using BSA/mBSA dosimetry, higher APRI scores were associated with worse survival (Supplementary Fig. 1A and 1C), whereas no survival differences between APRI groups were observed with partition model dosimetry (Supplementary Fig. 1B and 1D). These findings should be interpreted with caution, as dosimetry strategy was not randomly assigned and was strongly associated with tumour burden and spread in both cohorts (Supplementary Table 2).

We hypothesise that the negative survival results observed in large-scale HCC trials, such as SARAH and SIRveNIB [17, 18], could be related to the use of BSA/mBSA dosimetry, which was later assessed in a post-hoc analysis of the SARAH cohort showing that higher tumour-absorbed doses were associated with better OS [19]. Recent evidence from the DOSISPHERE-01 trial supports this, highlighting the benefits of partition model [20]. Notably, in our cohort median absorbed doses were similar between the BSA/mBSA and partition-model groups, despite the more diffuse disease pattern in the BSA/mBSA arm. This observation supports the hypothesis that intrahepatic dose distribution, rather than nominal dose magnitude alone, may be critical for treatment efficacy. Within the limitations of our sample size and observational design, the absence of APRI-related survival differences in the partition-model subgroup may suggest that personalised dosimetry could reduce the impact of underlying hepatic injury. However, this interpretation remains hypothesis-generating and may be influenced by residual confounding.

Importantly, although OS is a clinically meaningful endpoint, it is not treatment-specific and may be influenced by competing risks. Therefore, the present findings should be interpreted primarily as reflecting overall prognosis after TARE rather than direct treatment response to TARE itself. Radiologic and disease-control endpoints, such as mRECIST response and PFS, would provide complementary information.

In conclusion, the findings suggest that APRI may serve as a useful prognostic marker for OS after TARE in HCC and ICC. However, its association with OS should be interpreted as reflecting global post-treatment prognosis rather than a TARE-specific effect alone. Further analyses incorporating radiologic response and progression-based endpoints are warranted to better define the role of APRI.

## Supplementary Information

Below is the link to the electronic supplementary material.

Supplementary file1 (DOCX 233 KB)
