# Supplementary material for: Aspartate Aminotransferase to Platelet Index (APRI) as a Prognostic Factor for Overall Survival After TARE Y90 Resin Microspheres in Primary Liver Cancer Patients: Results from the European, Prospective Observational Study CIRT
Source: Cardiovasc Intervent Radiol. 2026 May 27;49(10):2064–71. doi: 10.1007/s00270-026-04483-7 (PMC13642421; doi:10.1007/s00270-026-04483-7)
Supplement: Supplementary file 1 [file 270_2026_4483_MOESM1_ESM.docx]

# Methods: supplementary information

## Statistical analysis

Data are presented as mean ± SD or median (IQR) for continuous variables and number (%) for categorical variables. Percentages are based on the whole cohort (n = 422 for HCC, n = 120 for ICC) unless otherwise indicated. APRI was calculated according to Wai et al. [7].

$$APRI=\frac{\frac{AST level (\frac{IU}{L})}{AST upper limit of normal (ULN)}}{Platelet counts (\frac{{10}^{9}}{L})}*100$$

Comparisons between groups were performed using the log-rank test (modified Peto-Peto). The median OS were calculated with their associated 95% confidence intervals (CIs). The group effect was calculated with a Cox proportional-hazards model with hazard ratio (HR) and 95% CIs.

HRs and 95% CIs in the univariable and multivariable analyses were calculated using the Cox proportional hazards regression model. Variable-associated p-values were determined with the Wald test, while overall model significance was assessed with the likelihood ratio test. Variables were initially selected for the multivariable model with a univariable p-value <0.1 and refined via backwards elimination. Cirrhosis and ascites were not considered for the model, due to their collinearity with APRI, and Child-Pugh was excluded due to missing data. A sensitivity analysis was performed to assess the effect of including ALBI in the multivariable models with respect to APRI.

To address potential confounding, propensity score matching was employed to balance covariates associated with overall survival. Importantly, matching was not based on variables linked to the exposure itself (i.e., APRI cut-off groups), but rather on those with a plausible influence on the outcome. Due to the inability of the matching algorithm to accommodate missing values, the dataset was correspondingly reduced. Covariate balance was assessed using standardised mean differences (SMD) and empirical cumulative distribution function (eCDF) statistics. A threshold of ±0.1 for SMD was considered acceptable, with eCDF mean and maximum values ideally approaching zero; minor deviations were tolerated if they improved the overall balance. In selected comparisons, matching with replacement was applied to preserve sample size and mitigate the loss of statistical power. The corresponding table outlines the number of observations retained and the effective sample size (ESS) per group.

Statistical analyses were performed using R version 4.4.3 (R Foundation, Vienna, Austria) with identical case report forms (eCRFs) and study procedures across studies.

# Results: supplementary information

## Propensity score analysis

Propensity score matching was performed separately for the ICC and HCC cohorts to reduce confounding and achieve balance between APRI groups. For ICC, matching was based on age, gender, ECOG performance status, tumour burden and prior hepatic procedures. Due to missing data, the sample size was reduced from 103 to 100 patients. Full matching achieved satisfactory covariate balance, with all standardised mean differences below ±0.1. The eCDF values confirmed adequate balance (eCDF mean = 0.02, max = 0.09). After matching, the ESS was 16.7 for APRI ≤0.4 and 67 for APRI >0.4, using full matching with replacement to preserve statistical power.

In ICC, APRI >0.4 remained significantly aligned with reduced overall survival in all models. The hazard ratio (HR) for APRI >0.4 increased from 2.07 (95% CI: 1.13–3.79, p=0.018) in the unweighted multivariable model to 2.55 (1.25–5.23, p=0.010) in the final weighted model after repeated backward elimination, suggesting that adjusting for baseline imbalance strengthened the predictive value of APRI. Tumour burden also remained an independent predictor of worse outcome in the weighted models, with uncountable nodules leading to an HR of 3.28 (1.57–6.86, p=0.002). The overall model fit did improve, with a concordance of 0.703 and R² = 0.263.

For the HCC cohort, matching was conducted using age, ECOG, prior hepatic procedures, and creatinine levels, with the sample reduced from 377 to 374 patients due to missing data. Full matching with probit linking again achieved excellent balance across covariates with an ESS of 62.3.

In the HCC analysis, APRI >0.7 was consistently associated with inferior survival. In the unweighted multivariable model, the HR was 1.44 (1.09–1.90, p=0.010), and remained significant in the weighted model after repeated elimination (HR = 1.46, 1.04–2.06, p=0.028). Main portal vein thrombosis also strongly predicted poor outcome (HR = 2.40, p=0.001). While the use of the partition model remained protective in univariate analyses, its effect was attenuated in multivariable modelling. Overall model performance improved after weighting, with concordance increasing to 0.672 and R² = 0.167.

In both cohorts, propensity score matching improved covariate balance without significant loss of sample size, and consistently confirmed APRI as an independent prognostic factor for survival.

# Limitations

# This study has several limitations. Firstly, despite its prospective character, this is an unplanned analysis potentially introducing a selection bias and residual confounding, despite the use of propensity score matching to adjust for key covariates. Second, while APRI was used as a surrogate for liver fibrosis, it may not fully capture the complexity of hepatic functional reserve, especially in the context of prior systemic therapy or mixed aetiologies of liver disease. Third, the choice of dosimetry method was not randomised but based on institutional practice and tumour extent, which could introduce allocation bias. Lastly, the high proportion of missing data for variables such as Child-Pugh may limit the interpretation of the results. Although the sample size was powerful enough for most subgroup analyses, some strata — particularly ICC patients with multiple prior chemotherapy lines — were small and may limit statistical power.

**Supplementary Table 1:** Disease and treatment characteristics

| Category | Subcategory | Cholangiocarcinoma | Hepatocellular Carcinoma |
| --- | --- | --- | --- |
| Total | | | |
|  |  | 103 | 377 |
| Cirrhosis | | | |
|  | n (%) | 103 (100%) | 377 (100%) |
|  | No | 90 (87.4%) | 112 (29.7%) |
|  | Yes | 13 (12.6%) | 265 (70.3%) |
| Ascites | | | |
|  | n (%) | 103 (100%) | 377 (100%) |
|  | No | 93 (90.3%) | 321 (85.1%) |
|  | Yes | 10 (9.7%) | 56 (14.9%) |
| Tumour Burden (nodules) | | | |
|  | n (%) | 103 (100%) | 377 (100%) |
|  | 1 | 35 (34%) | 126 (33.4%) |
|  | 2-5 | 23 (22.3%) | 122 (32.4%) |
|  | >5 | 17 (16.5%) | 65 (17.2%) |
|  | Uncountable | 28 (27.2%) | 64 (17%) |
| Liver tumour location | | | |
|  | n (%) | 103 (100%) | 377 (100%) |
|  | Bilobar | 58 (56.3%) | 131 (34.7%) |
|  | Left | 14 (13.6%) | 49 (13%) |
|  | Right | 31 (30.1%) | 197 (52.3%) |
| Portal vein thrombosis | | | |
|  | n (%) | 103 (100%) | 377 (100%) |
|  | Patent | 85 (82.5%) | 254 (67.4%) |
|  | Segmental Thrombosis | 10 (9.7%) | 77 (20.4%) |
|  | Lobar Thrombosis | 4 (3.9%) | 30 (8%) |
|  | Main Thrombosis | 4 (3.9%) | 16 (4.2%) |
| Lung shunt (%) | | | |
|  | n (%) | 102 (99%) | 369 (97.9%) |
|  | Median (IQR) | 5.0 (3.0 to 7.7) | 6.0 (3.6 to 9.1) |
| Concomiatant chemotherapy | | | |
|  | n (%) | 103 (100%) | 377 (100%) |
|  | No | 97 (94.2%) | 366 (97.1%) |
|  | Yes | 6 (5.8%) | 11 (2.9%) |
| Dose Methodology | | | |
|  | n (%) | 101 (98.1%) | 375 (99.5%) |
|  | BSA/mBSA | 64 (62.1%) | 205 (54.4%) |
|  | Partition Model | 37 (35.9%) | 170 (45.1%) |
| Prescribed activity (GBq) | | | |
|  | n (%) | 103 (100%) | 377 (100%) |
|  | Median (IQR) | 1.5 (1.1 to 1.8) | 1.4 (1.0 to 1.8) |
| *Abbreviations. IQR: interquartile range; BSA: body surface area.* | | | |

**Supplementary Figure 1:** Kaplan-Meier curves on the effect of dosimetry on survival

*Overall survival of ICC (A – B) and HCC (C – D) patients, treated using BSA/mBSA (A+ C) or the partition model (B + D).*

**Supplementary Table 2:** Tumour spread and dosimetry method

| HCC | |  | BSA/mBSA | Partition Model | p | |  |
| --- | --- | --- | --- | --- | --- | --- | --- |
| Total N (%) | | | 205 (54.7) | 170 (45.3) |  | |  |
| Tumour Spread (Location and number of nodules) | | | | | | |  |
|  | | Left 1 | 9 (4.4) | 13 (7.6) | <0.001 | |  |
|  | | Right 1 | 26 (12.7) | 70 (41.2) |  | |  |
|  | | Bilobar 1 | 5 (2.4) | 2 (1.2) |  | |  |
|  | | Left 2-5 | 3 (1.5) | 6 (3.5) |  | |  |
|  | | Right 2-5 | 32 (15.6) | 36 (21.2) |  | |  |
|  | | Bilobar 2-5 | 32 (15.6) | 13 (7.6) |  | |  |
|  | | Left >5 | 9 (4.4) | 1 (0.6) |  | |  |
|  | | Right >5 | 12 (5.9) | 5 (2.9) |  | |  |
|  | | Bilobar >5 | 33 (16.1) | 5 (2.9) |  | |  |
|  | | Left Uncountable | 4 (2.0) | 3 (1.8) |  | |  |
|  | | Right Uncountable | 8 (3.9) | 7 (4.1) |  | |  |
|  | | Bilobar Uncountable | 32 (15.6) | 9 (5.3) |  | |  |
| Tumour Burden (nodules) | | | | | | |  |
|  | | 1 | 40 (19.5) | 85 (50.0) | <0.001 | |  |
|  | | 2-5 | 67 (32.7) | 55 (32.4) |  | |  |
|  | | >5 | 54 (26.3) | 11 (6.5) |  | |  |
|  | | Uncountable | 44 (21.5) | 19 (11.2) |  | |  |
| Tumour Location | | | | | | |  |
|  | | Left | 25 (12.2) | 23 (13.5) | <0.001 | |  |
|  | | Right | 78 (38.0) | 118 (69.4) |  | |  |
|  | | Bilobar | 102 (49.8) | 29 (17.1) |  | |  |
| Absorbed Dose (Gy) | | | | | | |  |
|  | | Mean (SD) | 1112.3 (1986.0) | 958.4 (1646.4) | 0.435 | |  |
| Absorbed Dose (categorised) | | | | | | |  |
|  | | ≤400 | 91 (50.8) | 76 (45.5) | 0.377 | |  |
|  | | >400 | 88 (49.2) | 91 (54.5) |  | |  |
| ICC |  | | BSA/mBSA | Partition Model | | p | |
| Total N (%) | | | 64 (63.4) | 37 (36.6) | |  | |
| Tumour Spread (Location and number of nodules) | | | | | | | |
|  | Left 1 | | 2 (3.1) | 3 (8.1) | | 0.016 | |
|  | Right 1 | | 8 (12.5) | 11 (29.7) | |  | |
|  | Bilobar 1 | | 5 (7.8) | 5 (13.5) | |  | |
|  | Left 2-5 | | 1 (1.6) | 2 (5.4) | |  | |
|  | Right 2-5 | | 2 (3.1) | 5 (13.5) | |  | |
|  | Bilobar 2-5 | | 8 (12.5) | 5 (13.5) | |  | |
|  | Left >5 | | 3 (4.7) | 1 (2.7) | |  | |
|  | Right >5 | | 2 (3.1) | 1 (2.7) | |  | |
|  | Bilobar >5 | | 9 (14.1) | 0 (0.0) | |  | |
|  | Left Uncountable | | 2 (3.1) | 0 (0.0) | |  | |
|  | Right Uncountable | | 1 (1.6) | 1 (2.7) | |  | |
|  | Bilobar Uncountable | | 21 (32.8) | 3 (8.1) | |  | |
| Tumour Burden (nodules) | | | | | | | |
|  | 1 | | 15 (23.4) | 19 (51.4) | | <0.001 | |
|  | 2-5 | | 11 (17.2) | 12 (32.4) | |  | |
|  | >5 | | 14 (21.9) | 2 (5.4) | |  | |
|  | Uncountable | | 24 (37.5) | 4 (10.8) | |  | |
| Tumour Location | | | | | | | |
|  | Left | | 8 (12.5) | 6 (16.2) | | 0.005 | |
|  | Right | | 13 (20.3) | 18 (48.6) | |  | |
|  | Bilobar | | 43 (67.2) | 13 (35.1) | |  | |
| Absorbed Dose (Gy), lesions | | | | | | | |
|  | Mean (SD) | | 536.0 (797.1) | 559.7 (765.2) | | 0.887 | |
| Absorbed Dose (categorised) | | | | | | | |
|  | ≤300 | | 34 (55.7) | 15 (42.9) | | 0.316 | |
|  | >300 | | 27 (44.3) | 20 (57.1) | |  | |
| *Abbreviations. BSA: body surface area; SD: standard deviation; Gy: grey.* | | | | | | | |

**Supplementary Table 3:** Multivariable Analysis including ALBI in ICC

| OS |  | all | HR (univariable) | HR (multivariable) |
| --- | --- | --- | --- | --- |
| Tumour burden (nodules) | | | | |
|  | 1 | 35 (35.0) | - | - |
|  | 2-5 | 23 (23.0) | 2.38 (1.15-4.94, p=0.020) | 1.70 (0.72-4.02, p=0.226) |
|  | >5 | 16 (16.0) | 3.68 (1.78-7.63, p<0.001) | 3.36 (1.57-7.19, p=0.002) |
|  | Uncountable | 26 (26.0) | 2.56 (1.33-4.94, p=0.005) | 3.20 (1.50-6.81, p=0.003) |
| Portal Vein Occlusion | | | | |
|  | Patent | 83 (83.0) | - | - |
|  | Segmental Thrombosis | 9 (9.0) | 0.98 (0.42-2.28, p=0.961) | 0.87 (0.36-2.11, p=0.763) |
|  | Lobar Thrombosis | 4 (4.0) | 0.64 (0.16-2.65, p=0.542) | 0.60 (0.30-1.20, p=0.148) |
|  | Main Thrombosis | 4 (4.0) | 4.23 (1.45-12.31, p=0.008) | 5.74 (2.34-14.10, p<0.001) |
| ALBI grade | | | | |
|  | A1 | 51 (56.7) | - | - |
|  | A2 | 38 (42.2) | 0.92 (0.54-1.54, p=0.740) | 0.69 (0.39-1.22, p=0.203) |
|  | A3 | 1 (1.1) | 1.67 (0.23-12.31, p=0.615) | 3.46 (1.27-9.42, p=0.015) |
| APRI | | | | |
|  | ≤0.4 | 33 (33.0) | - | - |
|  | >0.4 | 67 (67.0) | 2.23 (1.26-3.95, p=0.006) | 2.58 (1.42-4.71, p=0.002) |
| Creatinine (mg/dl) | | | | |
|  | Mean (SD) | 1.0 (1.0) | 1.20 (1.00-1.44, p=0.053) | 1.13 (0.99-1.29, p=0.063) |
| Number in dataframe = 100, Number in model = 90, Missing = 10, Number of events = 61, Concordance = 0.717 (SE = 0.038), R-squared = 0.315 (Max possible = 0.994), Likelihood ratio test = 34.029 (df = 10, p = 0.000) | | | | |

**Supplementary Table 4:** Multivariable Analysis including ALBI in HCC

| OS |  | all | HR (univariable) | HR (multivariable) |
| --- | --- | --- | --- | --- |
| Extra-hepatic disease at first treatment | | | | |
|  | No | 339 (90.6) | - | - |
|  | Yes | 35 (9.4) | 1.92 (1.27-2.90, p=0.002) | 1.44 (0.92-2.25, p=0.109) |
| Liver tumour location | | | | |
|  | Bilobar | 129 (34.5) | - | - |
|  | Left | 49 (13.1) | 0.54 (0.35-0.84, p=0.006) | 0.55 (0.28-1.05, p=0.072) |
|  | Right | 196 (52.4) | 0.50 (0.37-0.66, p<0.001) | 0.53 (0.34-0.81, p=0.004) |
| Portal Vein | | | | |
|  | Patent | 252 (67.4) | - | - |
|  | Segmental Thrombosis | 76 (20.3) | 1.08 (0.77-1.51, p=0.663) | 1.40 (0.94-2.08, p=0.095) |
|  | Lobar Thrombosis | 30 (8.0) | 1.42 (0.86-2.32, p=0.166) | 1.31 (0.67-2.57, p=0.426) |
|  | Main Thrombosis | 16 (4.3) | 2.46 (1.39-4.34, p=0.002) | 2.10 (1.16-3.83, p=0.015) |
| Dose Methodology | | | | |
|  | BSA/mBSA | 202 (54.3) | - | - |
|  | Partition Model | 170 (45.7) | 0.52 (0.39-0.69, p<0.001) | 0.67 (0.42-1.06, p=0.087) |
| ALBI grade | | | | |
|  | A1 | 128 (36.6) | - | - |
|  | A2 | 208 (59.4) | 1.53 (1.14-2.05, p=0.005) | 1.55 (1.00-2.40, p=0.050) |
|  | A3 | 14 (4.0) | 4.67 (2.44-8.92, p<0.001) | 4.27 (1.83-9.96, p=0.001) |
| APRI | | | | |
|  | ≤0.7 | 166 (44.4) | - | - |
|  | >0.7 | 208 (55.6) | 1.42 (1.09-1.86, p=0.010) | 1.21 (0.80-1.83, p=0.361) |
| Number in dataframe = 374, Number in model = 349, Missing = 25, Number of events = 209, Concordance = 0.699 (SE = 0.022), R-squared = 0.207 (Max possible = 0.998), Likelihood ratio test = 81.149 (df = 10, p = 0.000) | | | | |
